# Supplementary material for: Are people really less moral in their foreign language? Proficiency and comprehension matter for the moral foreign language effect in Russian speakers
Source: PLoS One. 2023 Jul 10;18(7):e0287789. doi: 10.1371/journal.pone.0287789 (PMC10332622; doi:10.1371/journal.pone.0287789)
Supplement: S1 Appendix — (DOCX) [file pone.0287789.s001.docx]

**Appendix A**

**Process Dissociation Calculations**

To calculate the deontology and utilitarian PD parameters one must examine harm acceptance and rejection responses to both congruent and incongruent dilemmas. Harmful action maximizes overall outcomes for incongruent, but not congruent, dilemmas. Therefore, responses consistent with utilitarianism will entail accepting harm on incongruent dilemmas, when harm maximizes outcomes, but rejecting harm on congruent dilemmas where harm arguably does not maximize outcomes. Conversely, responses consistent with deontology will entail rejecting harm in all cases.

Consider the processing tree depicted in Fig S1. The top path illustrates the case where responses are consistent with utilitarianism: rejecting harm for congruent dilemmas but accepting harm for incongruent dilemmas. The second path illustrates the case where responses are consistent with deontology: rejecting harm for both congruent and incongruent dilemmas. Finally, the bottom path represents the case where responses are consistent with neither utilitarianism nor deontology; this case entails accepting harm for both congruent and incongruent dilemmas.

**Fig S1.** Processing tree illustrating the components underlying responses to congruent and incongruent moral dilemmas.


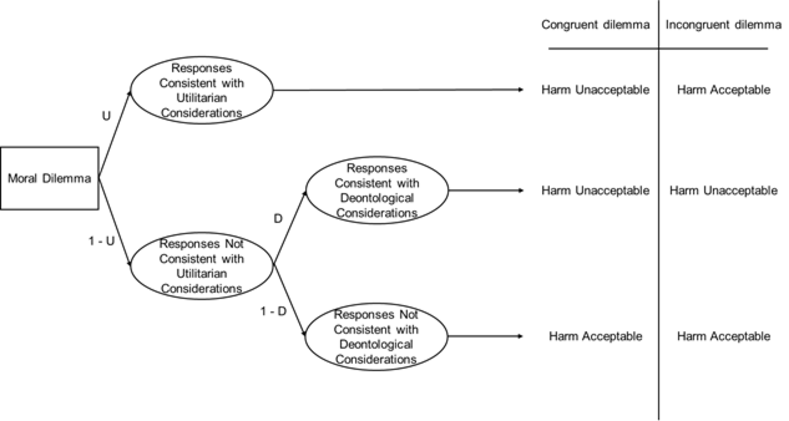


The two columns on the right side of the figure allow for working backward to determine which cases led participants to judge harm as acceptable or unacceptable for congruent and incongruent dilemmas. For congruent dilemmas, harm is unacceptable when responses are consistent with either utilitarianism, *U*, or deontology, *(1 – U) × D*. Conversely, harm is acceptable on congruent dilemmas when responses are consistent with neither utilitarianism nor deontology, *(1 – U) × (1 – D)*. For incongruent dilemmas, harm is unacceptable when responses are consistent with deontology, *(1 – U) × D*. Conversely, harm is acceptable either when consistent with utilitarianism, *U*, or consistent with neither utilitarianism nor deontology, *(1 – U) × (1 – D)*.

Combining these cases allows for algebraically representing the probability of a particular judgment. For example, the probability of judging harm as unacceptable for congruent dilemmas is represented by the case where responses are consistent with either utilitarianism or deontology:

Eq. (A.1): $p(unacceptable | congruent)=U + [(1 - U) \times D]$

Conversely, the probability of judging harm as acceptable in congruent dilemmas is represented by the case that responses are consistent with neither utilitarianism or deontology:

Eq. (A.2): $p(acceptable | congruent)=(1 - U) \times(1 - D)$

For incongruent dilemmas, the probability of judging harm as unacceptable is represented by the case responses are consistent with deontology:

Eq. (A.3): $p(unacceptable | incongruent)=(1 - U) \times D$

Conversely, the probability of judging harm as acceptable for incongruent dilemmas is represented by the cases that consistent with utilitarianism, or consistent with neither utilitarianism nor deontology:

Eq. (A.4): $p(acceptable | incongruent)=U + [(1 - U) \times(1 - D)]$

By algebraically representing the probabilities of accepting and rejecting harm in congruent and incongruent dilemmas, one can enter a participants’ pattern of actual responses across multiple congruent and incongruent dilemmas, and algebraically combine these equations to solve for parameters estimating deontological (*D*) and utilitarian (*U*) inclinations underpinning their responses. By including Equation A.3 into Equation A.1, the latter can be solved for *U*, leading to the following formula:

Eq. (A.5): $U=p(unacceptable | congruent) - p(unacceptable | incongruent)$

By including the calculated value for *U* in Equation A.3, this equation can be solved for *D*, leading to the following formula:

Eq. (A.6): $D=p(unacceptable | incongruent) / (1 - U)$

Together, these formulas provide a method of independently estimating the strength of deontological and utilitarian inclinations underlying conventional moral dilemma judgments.
