# Supplementary material for: Are people really less moral in their foreign language? Proficiency and comprehension matter for the moral foreign language effect in Russian speakers
Source: PLoS One. 2023 Jul 10;18(7):e0287789. doi: 10.1371/journal.pone.0287789 (PMC10332622; doi:10.1371/journal.pone.0287789)
Supplement: S2 Appendix — (DOCX) [file pone.0287789.s002.docx]

**Appendix B**

**Linguistic Background Questionnaire**

1. How old are you?
2. What is your gender?
3. What is your first language?
4. Do you have a speech or hearing impediment?
5. If you have a speech or hearing impediment, please, provide details below.
6. Are you right-handed or left-handed?
7. How old were you when you first started learning English?
8. How many years total have you studied English?
9. How many hours per week did you have English classes in:

|  |  | 0 hrs. | 1 hr. | 2 hrs. | 3 hrs. | 4 hrs. | 5 hrs. and more |
| --- | --- | --- | --- | --- | --- | --- | --- |
| 1 | Elementary school |  |  |  |  |  |  |
| 2 | Middle school |  |  |  |  |  |  |
| 3 | High school |  |  |  |  |  |  |
| 4 | Private lessons |  |  |  |  |  |  |
| 5 | English language courses |  |  |  |  |  |  |

1. Did you take English language courses?
2. If you took English language courses, your teacher was

- Russian native speaker
- English native speaker

1. Have you ever lived in an English-speaking country?
2. If you have lived in an English-speaking country, please indicate

- The name of the country
- How many days/weeks/months/years you stayed there
- When you went there

1. If you have lived in an English-speaking country, did you stay with English native speakers?
2. How long did you stay with English native speakers?
3. If you stayed with English native speakers, how often did you interact with them in English?

- We only interacted with them if it was absolutely necessary.
- We interacted sometimes
- We interacted fairly often
- We interacted very often

1. Do you have relatives or close friends whose native language is English?
2. If you have relatives or close friends whose native language is English, how often do you interact with them in English?

- We don’t interact in English.
- We very seldom interact in English – once or twice a year.
- We sometimes interact in English – several times a year.
- We interact in English fairly often – at least once a month.
- We interact in English often – several times a month.
- We interact in English very often – (almost) every day.

1. Please, rate your English proficiency:

|  |  | Beginner | Low-intermediate | Intermediate | Upper- intermediate | Advanced |
| --- | --- | --- | --- | --- | --- | --- |
| 1 | Reading |  |  |  |  |  |
| 2 | Speaking |  |  |  |  |  |
| 3 | Writing |  |  |  |  |  |
| 4 | Listening |  |  |  |  |  |

1. What languages beside English have you studied? Please, name the languages, how many years you have studied them, and what your proficiency is in those languages (beginner, intermediate, advanced).
