# Supplementary material for: Are people really less moral in their foreign language? Proficiency and comprehension matter for the moral foreign language effect in Russian speakers
Source: PLoS One. 2023 Jul 10;18(7):e0287789. doi: 10.1371/journal.pone.0287789 (PMC10332622; doi:10.1371/journal.pone.0287789)
Supplement: S3 Appendix — (DOCX) [file pone.0287789.s003.docx]

**Appendix C**

**MELICET Test Adapted**

**Participant:**

**SECTION 1: Grammar**

**INSTRUCTIONS:** Choose the word or phrase that best completes the conversation.

1. “What time will we arrive in San Francisco?”

“I’m not sure, because I don’t know __________ from here.”

choose one (a) it is how far, b) how far is it, c) how far it is, d) how is it far)

2. “Did George enter the photography contest?”

“No, but if he had, I think he __________.”

choose one (a) would have won, b) had won, c) would won, d) will have won)

3. “What’s the matter?”

“I feel __________ out.”

choose one (a) tire, b) tiring, c) tired, d) being tired)

4. “May I bring you a cup of tea?”

“I prefer coffee __________ tea.”

choose one (a) to, b) than, c) rather, d) instead)

5. “Have you ever gone to Tahiti?”

“No, but I have __________ for a long time.”

choose one (a) wanted, b) been wanted, c) wanting to, d) been wanting to)

6. “Will you come to my party on Saturday?”

“__________ I’d like to, I can’t.”

choose one (a) The same as, b) As much as, c) So much that, d) More than)

7. “Don’t forget to pay the rent tomorrow!”

“Please remind __________ in the morning.”

choose one (a) me it, b) me of it, c) it to me, d) it me)

8. “Susan plays the piano very well.”

“__________ that, she’s an excellent singer.”

choose one (a) As well, b) But also, c) Not only, d) In addition)

9. “Which chair should I take?”

“The __________ over there.”

choose one (a) bright green folding one, b) bright folding green, c) one bright green folding, d) one bright folding green)

10. “Mark isn’t very smart, is he?”

“Actually, he’s smarter than he __________ to be.”

choose one (a) might seem, b) seem, c) is seeming, d) is seemed)

11. “What do you think of American football?”

“I think it’s __________ sport.”

choose one (a) very dangerous, b) very dangerous a, c) too dangerous a, d) too dangerous)

12. “What shall we do about this problem?”

“John suggests __________ a meeting.”

choose one (a) of calling, b) that call, c) that will call, d) calling)

13. “Where did you get those curtains?”

“My wife made them __________ an old tablecloth.”

choose one (a) from out, b) from in, c) out from, d) out of)

14. “Do you like sugar in your coffee?”

“Yes, __________ better.”

choose one (a) the more the, b) more the, c) the more, d) more)

15. “Why did John refuse to pay for his dinner?”

“Because __________ two hours by the time he was served.”

choose one (a) he’s been waiting, b) he’s been waited, c) he’d been waiting, d) he’d been waited)

16. “When is the meeting going to begin?”

“__________ Fred comes, we can get started.”

choose one (a) Then, b) Until, c) Once, d) At)

17. “Does John have a lot of accidents at work?”

“Yes. He isn’t __________ he should be.”

choose one (a) as careful work, b) as careful as a worker, c) as careful worker, d) as careful a worker as)

18. “Did David enter the writing contest?”

“Yes, he thinks he has __________.”

choose one (a) a chance to win, b) a chance of win, c) a chance win, d) the chance to win)

19. “Does Barbara have a difficult job?”

“Yes. She is responsible __________ many important decisions.”

choose one (a) her making, b) for making, c) to make, d) of making)

20. “You gave me the wrong amount of money.”

“How __________? I gave you what you asked for.”

choose one (a) can be that, b) can that be, c) can be, d) that can be)

21. “Will Bill’s report be ready by Friday?”

“No, I don’t think he __________ it by then.”

choose one (a) has finished, b) will have finished, c) finishes, d) will be finished)

22. “When will this paint be dry?”

“Not long. This is very __________ paint.”

choose one (a) fast to dry, b) fast drying, c) dry fast, d) fast dry)

23. “Does Sue like circuses?”

“Yes, the clowns always make __________.”

choose one (a) her laughing, b) she laughs, c) her laugh, d) her to laugh)

24. “Did you do well on the history test?”

“No. I studied all night __________ failed.”

choose one (a) still yet, b) even though, c) although, d) but still)

25. “How do those shoes fit?”

“My feet are too big __________ them.”

choose one (a) for wear, b) that to wear, c) to wear, d) that I can’t wear)

26. “Do Mary’s children help with the housework?”

“Yes, if she asks __________.”

choose one (a) it them, b) them to do, c) them to, d) them for)

27. “Where’s the box I asked for?”

“Over there, __________ on the table.”

choose one (a) seated, b) sitting, c) sat, d) seating)

28. “Let’s plan a picnic for Saturday.”

“__________ it rains?”

choose one (a) What if, b) Nevertheless, c) Except, d) In spite of)

29. “Is Lynn going to buy a new suit?”

“Yes, she’s looking for a suit like __________.”

choose one (a) your wool one, b) one wool of yours, c) wool one of yours, d) one of your wool)

30. “That movie isn’t very good.”

“Just wait. The best part __________.”

choose one (a) has come yet, b) is yet coming, c) is yet to come, d) come yet)

**SECTION 2: Cloze**

**INSTRUCTIONS:** Read the passage, then select the word which best fills the blank in both grammar and meaning.

Color is such a constant part of our environment that we tend to ignore its messages.

Many people with perfect vision suffer 31 (of, from, such, like) a sort of cultural color blindness. But 32 (that, other, even, have) unnoticed color influences feelings as well. 33 (Many, Some, Lot, Reports) of experiments with both infants and 34 (colors, adults, also, muscles) indicate that blue light tends to 35 (lessen, create, release, increase) activity and produce a state of restfulness. 36 (Even, But, As, The) more tense a person is, the 37 (more, color, light, worse) blue will act as a tranquilizer. Red, 38 (in, is, on, affects) the contrary, excites the nervous system, 39 (and, so, suppose, imagine) that if this page were printed 40 (with, on, as, in) red paper, electrodes attached to your skin 41 (would, will, and, to) show a definite increase in muscle 42 (intensity, system, naturally, tension) , restlessness, and eye movements compared with 43 (eye, its, your, their) reactions to the white page. Studies 44 (were, have, which, nevertheless) found that patients in hospital rooms 45 (colored, see, painted, on) red or other bright colors require 46 (more, much, the, special) attention from nurses than patients in 47 (blue, not, rooms, which) painted in more subdued colors. Furthermore, 48 (studies, result, teachers, it) has been found that school children 49 (show, will, are, were) more alert and learn faster in 50 (brightly, red, blue, subdued) painted rooms. However, this is unfortunately accompanied by an increase in restlessness and noisiness.

[Adapted from MELICET test]
