# Supplementary material for: Are people really less moral in their foreign language? Proficiency and comprehension matter for the moral foreign language effect in Russian speakers
Source: PLoS One. 2023 Jul 10;18(7):e0287789. doi: 10.1371/journal.pone.0287789 (PMC10332622; doi:10.1371/journal.pone.0287789)
Supplement: Attachment — Submitted filename: Response to Reviewers.docx [file pone.0287789.s004.docx]

**COMMENT**

Reviewer #2: This paper shows a valuable contribution to the existing literature and knowledge in terms of introduction, style of writing, and methodology. Yet, there are minor typos that should be corrected.
The manuscript should be checked for minor language issues and the researchers need to mention to some tables in the explanations so that they are clearer and easier to be followed. Furthermore, The discussion of the study needs to be compared with more relevant new studies in relation to that period such as:
Liqaa Habeb Al-Obaydi (2021): Humanistic learning elements in a blended
learning environment: a study in an EFL teaching context, Interactive Learning Environments, DOI:
10.1080/10494820.2021.1919717
Finally,it still needs thorough proofreading and editing to polish its content, language, and APA style.
The manuscript looks much better. Thank you.

**RESPONSE**

We thank the reviewer for these encouraging words. We went through the manuscript to correct any remaining typos in the text and references and eliminated some phrases with different coloured text—see enclosed version with track changes.

We now mention tables in the discussion, line 478, line 490, and line 541, in addition to the results.

We also now cite the mentioned paper as follows: Finally, it may be that the learning conditions under which people encounter their FL influence responses, such as humanistic learning environments [84].
